# Association of serum interleukin-2 with severity and prognosis in hospitalized patients with community-acquired pneumonia: a prospective cohort study

**DOI:** 10.1007/s11739-024-03699-0

**Published:** 2024-07-05

**Authors:** Feng-Min Zhu, Juan Xu, Qi-Yuan He, You-Peng Deng, Ming-Yan Liu, Ying Liu, Jing Sun, Hui Zhao, Lin Fu, Jin Yang

**Affiliations:** 1 Department of Respiratory and Critical Care Medicine, The Second Affiliated Hospital of Anhui Medical University, Furong Road No 678, Hefei, 230601 Anhui China; 2 Institute of Respiratory Diseases, The Second Affiliated Hospital of Anhui Medical University, Hefei, 230601 Anhui China

**Keywords:** Community-acquired pneumonia, IL-2, CAP severity score, Biomarker

## Abstract

The prior studies have shown that interleukin-2 (IL-2) exerts important roles in the pathological and physiological processes of lung diseases. However, the role of IL-2 in community-acquired pneumonia (CAP) is still uncertain. Through a prospective cohort study, our research will explore the correlations between serum IL-2 levels and the severity and prognosis in CAP patients. There were 267 CAP patients included. Blood samples were obtained. Serum IL-2 were tested by enzyme-linked immunosorbent assay (ELISA). Demographic traits and clinical characteristics were extracted. Serum IL-2 were gradually elevated with increasing severity scores in CAP patients. Correlation analyses revealed that serum IL-2 were connected with physiological parameters including liver and renal function in CAP patients. According to a logistic regression analysis, serum IL-2 were positively correlated with CAP severity scores. We also tracked the prognostic outcomes of CAP patients. The increased risks of adversely prognostic outcomes, including mechanical ventilation, vasoactive agent usage, ICU admission, death, and longer hospital length, were associated with higher levels of IL-2 at admission. Serum IL-2 at admission were positively associated with severe conditions and poor prognosis among CAP patients, indicated that IL-2 may involve in the initiation and development of CAP. As a result, serum IL-2 may be an available biomarker to guide clinicians in assessing the severity and determining the prognosis of CAP.

**Supplementary Information:**

The online version contains supplementary material available at 10.1007/s11739-024-03699-0.

## Introduction

Community-acquired pneumonia (CAP) is a condition characterized by inflammation of the pulmonary tissue resulting from an infection contracted outside of hospital settings, namely within the community. Additionally, it ranks among the leading causes of death globally. Although it caused a large number of deaths, it is still not considered by the public as an issue that needs attention [1–5]. For CAP, the physician can diagnose by the main clinical manifestations and ancillary tests, but in many patients, the presentation of pneumonia may be atypical non-respiratory symptoms such as fatigue, muscle aches, diarrhea, etc. [3]. These atypical pneumonias may be overlooked by clinicians. Although the clinically diagnostic criteria and severity scores can guide the diagnosis and determine the severity of CAP, the subjectivity of clinical symptoms and prolonged pathogen cultivation may sometimes hinder accurate assessment of the severity and prognosis of CAP timely. Therefore, in order to reduce the progression of mild pneumonia to severe pneumonia, as well as the mortality, a characteristic biomarker is needed to aid in early assessment.

Interleukin-2 (IL-2), a common cytokine, which is mainly produced in the human body by helper T cells 1, and it possesses the capability to facilitate cellular growth and differentiation, as well as the secretion of cytokines, and directly or indirectly participates in cellular and humoral immune regulation [6]. The previous researches have indicated that numerous inflammation-mediated disorders, including Crohn's disease, autoimmune arthritis, obesity-related metabolic inflammation, etc., have been related with IL-2. [7–11]. Recent surveys have identified the correlations between IL-2 and several pulmonary diseases [12–18].

Though IL-2 plays a significant role in pulmonary diseases, it is unclear whether IL-2 is associated with CAP. Previous studies have shown that there are large numbers of recruited neutrophils in the lungs of pneumonia patients [19]. IL-2 can bind to the interleukin-2 receptor β and γ chains on human neutrophils to induce pulmonary edema [20, 21]. Therefore, we hypothesized that IL-2 may participate in the pathophysiologic process of CAP. This prospective cohort study sought to explore the correlations between serum IL-2 levels and the severity as well as the prognosis of CAP patients.

## Methods

### Subjects

From August 2021 to September 2022, this research was performed in the Second Affiliated Hospital of Anhui Medical University. Every patient fulfilled the following diagnostic criteria: (1) occurred in the community; (2) older than 18 years of age; (3) new or progressive infiltrative lesions of the lungs; (4) met one of the following symptoms: temperature > 38℃, respiratory symptoms such as coughing and sputum, signs of solid changes in the lungs on percussion and wet rales on auscultation, elevated white blood cell count [22, 23]. The exclusion criteria were: (1) pregnant women; (2) hospitalized for more than 2 weeks within 3 months due to illness; (3) treatment with intravenous antibiotics, antiviral drugs, glucocorticoid in the last week; (4) accompanied with other respiratory diseases such as lung malignancy, COPD, bronchiectasis; (5) accompanied with autoimmune diseases [24–26]. All 288 hospitalized patients who agreed to participate in this study were recruited. Twelve serum samples were lost and 9 patients with incomplete information were later excluded and there were 267 hospitalized patients enrolled in total (Supplemental Fig. 1). Additionally, in order to evaluate the level of IL-2 in healthy volunteers, healthy participators from the Physical Examination Center of the Second Affiliated Hospital of Anhui Medical University were collected. Every control subject was matched with one CAP patients in accordance with age and sex. The control groups were without respiratory system diseases such as CAP, COPD, asthma, lung cancers, and etc. Lastly, 267 eligible control subjects were selected and enrolled. Demographic characteristics and clinical information were gathered from the electronic medical record system. The related prognostic outcomes were tracked-up. The primary outcomes was mortality, the secondary outcomes were ICU admission, mechanical ventilation, vasoactive drugs and longer hospital stays among CAP inpatients. Before starting to use any antibiotics, serum samples were collected from each patient. Routine blood and routine biochemical tests were performed. In order to assess the extent of pneumonia severity, various severity scores have been utilized.

**Fig. 1:**
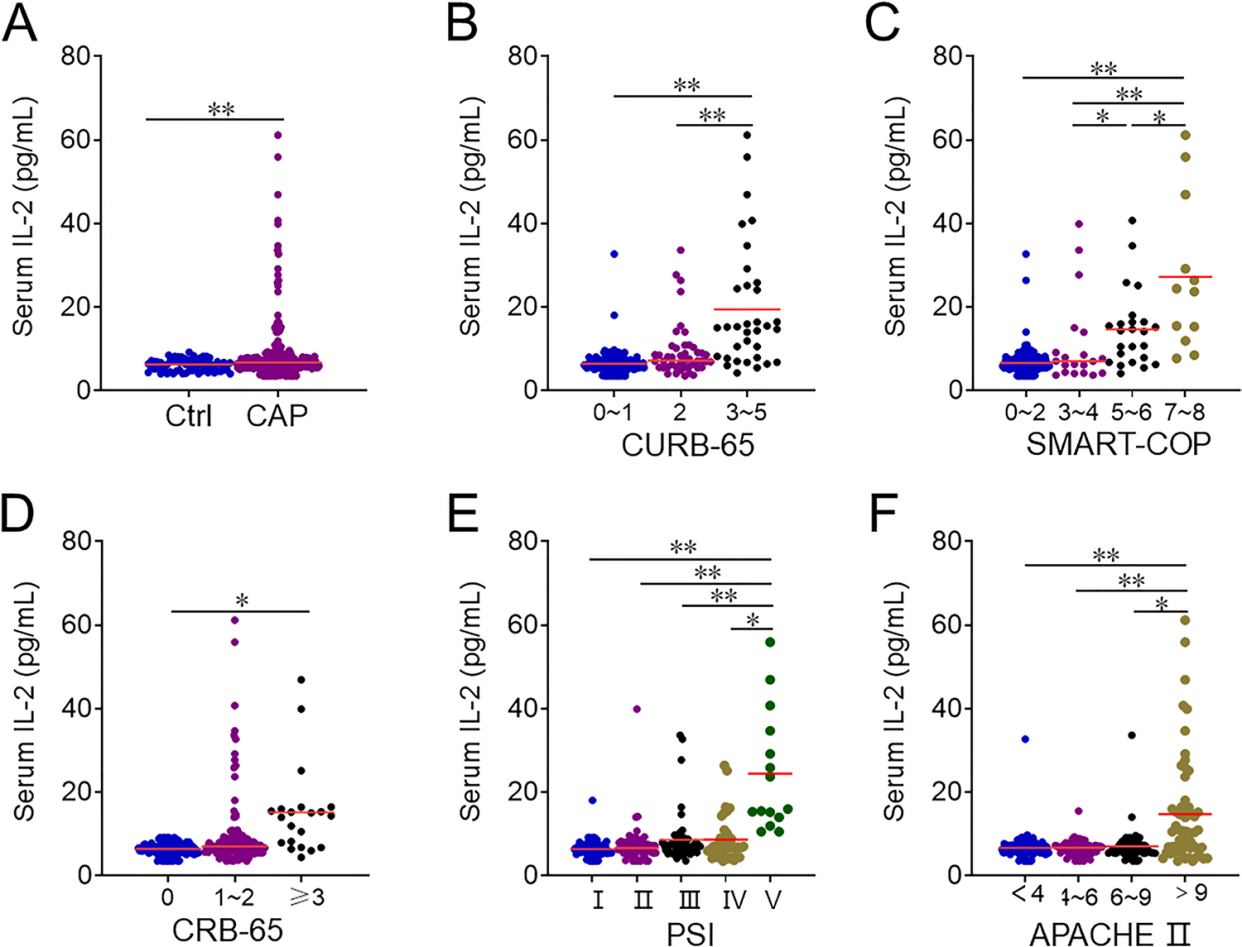
Serum IL-2 levels in CAP patients with different severity. The levels of serum IL-2 were measured through ELISA. **A** Serum IL-2 levels in healthy individuals and CAP patients. **B**–**F** The levels of serum IL-2 in CAP cases with different severity scores. **B** CURB-65 score. **C** SMART-COP score. **D** CRB-65 score. **E** PSI score. **F** APACHE II score. *P < 0.05, **P < 0.01

### Enzyme-linked immunosorbent assay (ELISA)

Fasting blood was collected and centrifuged at 6 a.m. on the second day after admission. Serum IL-2 were detected via ELISA at a unified time. IL-2 (CSB-E04626h) ELISA kits were from Cusabio in Wuhan, China. (https://www.cusabio.com/). All ELISA techniques were carried out in accordance with the earlier studies [27, 28].

### Statistical analysis

Demographic characteristics and laboratory parameters were represented by the median or mean. Categorical variables are represented by frequencies (percentages). To examine differences in characteristics between groupings, ANOVA or chi-square tests were employed. The relationships between clinical parameters and serum IL-2 were investigated using Spearman or Pearson correlative analyses and linear regression models. Linear and logistic regression models were utilized to determine the correlation between the levels of serum IL-2 and the severity of CAP. The association between prognostic outcomes and serum IL-2 was investigated using mixed logistic regression models. The presence of statistical significance was observed when the *P*-value was found to be less than 0.05 in the conducted research.

## Results

### Demographic and clinical information

Among all CAP patients, the median respiratory rate was 19.8 breaths per minute and the median oxygen saturation (room air) was 94.8% (Table 1). CAP patients were divided into three groups based on the tertiles of serum IL-2: tertile 1 (T1) with IL-2 below 5.86 pg/mL, tertile 2 (T2) with IL-2 ranging from 5.86 to 7.23 pg/mL, and tertile 3 (T3) with IL-2 above 7.23 pg/mL. As appeared in Table 1, of these, 157 were men, accounting for 58.8%. The median BMI was 22.3 kg/m^2^. In addition, the mean of systolic blood pressure was 125.2 mmHg, the average of diastolic blood pressures was 75.2 mmHg. Moreover, microbiological diagnosis was analyzed. Among all CAP patients, 108 (40.4%) cases were infected with *streptococcus pneumonia*, 13 (4.9%) cases with *staphylococcus aureus*, 18 (6.7%) cases with *legionella pneumophila*, 44 (16.1%) cases with other atypical pathogens. Moreover, 10 (3.7%) subjects with *respiratory virus*, 9 (3.4%) subjects with *pseudomonas aeruginosa*, 6 (2.2%) subjects with *enterobacteriaceae*, and 60 (22.5%) with other etiologies. Our study also analyzed the comorbid conditions in patients with CAP. In Table 1, 72 patients had hypertension (27.0%), 30 patients had diabetes mellitus (11.2%), 26 patients had the past history of cerebral infarction (9.7%), 15 patients had coronary heart disease (5.6%), and only 4 cases had chronic bronchitis (1.5%). Some serological markers were measured in all patients, such as procalcitonin (PCT), D- dimer, tumor necrosis factor-α (TNF-a), C-reaction protein (CRP), interleukin-6 (IL-6), etc. There was no distinction in age, gender, BMI, heart rate, blood pressure, body temperature, hypertension, diabetes mellitus, coronary artery disease, and bronchitis among CAP patients in three groups. Whereas the respiratory rate, PCT, D-dimer, TNF-α, and IL-6 increased with rising serum IL-2, and oxygen saturation decreased with growing IL-2 (Table 1). In addition, the CAP severity scores went up consistently as the increased serum IL-2 levels (Table 1).

**Table 1:** Demographic characteristics of participators at baseline

Characteristic	All participators	Tertile of serum IL-2					*P*
		Tertile 1 (< 5.86 pg/mL)	Tertile 2 (5.86 ~ 7.23 pg/mL)			Tertile 3 (> 7.23 pg/mL)	
N	267	89	89			89	
Age, years	58.9 ± 1.12	60.3 ± 1.90	53.5 ± 2.06			62.9 ± 1.75	0.404
Male, n (%)	157 (58.8)	51 (57.3)	51 (57.3)			55 (61.8)	0.781
BMI	22.3 ± 0.30	22.7 ± 0.57	21.8 ± 0.41			22.6 ± 0.49	0.073
*Streptococcus pneumonae*, n (%)	108 (40.4)	37 (41.6)	33 (37.1)			38 (42.7)	0.784
*Staphylococcus aureus*, n (%)	13 (4.9)	3 (3.4)	4 (4.5)			6 (6.7)	0.682
*Legionella pneumophila*, n (%)	18 (6.7)	6 (6.7)	5 (5.6)			7 (7.9)	0.953
*Pseudomonas aeruginosa*, n (%)	9 (3.4)	2 (2.2)	3 (3.4)			4 (4.5)	0.912
*Enterobacteriaceae*, n (%)	6 (2.2)	1 (1.1)	2 (2.2)			3 (3.4)	0.874
Other atypical pathogens, n (%)	43 (16.1)	15 (16.9)	13 (14.6)			15 (16.9)	0.934
*Respiratory virus*, n (%)	10 (3.7)	2 (2.2)	4 (4.5)			4 (4.5)	0.779
Others, n (%)	60 (22.5)	17 (19.1)	20 (22.5)			23 (25.8)	0.763
Heart rate (beats per min)	87.0 (78.0, 100.0)	87.0 (76.0, 100.0)	90.0 (77.0, 103.0)			86.0 (80.0, 98.0)	0.134
Respiratory rate (breaths per min)	19.8 ± 0.22	19.5 ± 0.30	19.5 ± 0.26			20.4 ± 0.48	**0.005**
Oxygen saturation (%)	94.8 ± 0.56	96.1 ± 0.33	96.2 ± 0.31			92.1 ± 1.56	**0.002**
Temperature (℃)	36.8 ± 0.06	36.7 ± 0.07	36.7 ± 0.06			36.8 ± 0.09	0.153
Systolic pressure (mmHg)	125.2 ± 1.19	127.1 ± 1.89	121.1 ± 1.98			127.2 ± 2.24	0.135
Diastolic pressure (mmHg)	75.2 ± 0.70	75.9 ± 1.29	75.4 ± 1.23			74.3 ± 1.22	0.464
Hypertension, n (%)	72 (27.0)	24 (27.0)	23 (25.8)			25 (28.1)	0.945
Diabetes mellitus, n (%)	30 (11.2)	9 (10.1)	10 (11.2)			11 (12.4)	0.893
Cerebral infarction, n (%)	26 (9.7)	4 (4.5)	6 (6.7)			16 (18.0)	**0.005**
Coronary heart disease, n (%)	15 (5.6)	5 (5.6)	5 (5.6)			5 (5.6)	1.000
Bronchitis, n (%)	4 (1.5)	0	2 (2.2)			2 (2.2)	0.362
Procalcitonin (ng/L)	0.07 (0.03, 0.49)	0.07 (0.02, 0.39)	0.07 (0.02, 0.48)			0.11 (0.04, 0.75)	**0.003**
D-dimer (mg/L)	1.7 ± 0.14	2.0 ± 0.26	1.3 ± 0.19			1.8 ± 0.27	**0.046**
Tumor necrosis factor alpha (pg/mL)	7.6 (4.4, 23.1)	5.1 (4.9, 19.6)	6.1 (4.4, 28.0)			8.7 (4.1, 25.8)	**0.046**
C-reactive protein (mg/L)	59.7 (7.7, 142.7)	57.9 (6.9, 138.4)	59.7 (7.9, 140.9)			63.8 (9.1, 148.6)	0.344
Interleukin-6 (pg/mL)	15.0 (4.1, 44.6)	10.8 (1.9, 36.4)	13.9 (4.8, 45.5)			21.9 (5.8, 70.9)	**0.019**
CURB-65	1.2 ± 0.05	0.8 ± 0.10	0.7 ± 0.10			1.5 ± 0.14	**0.000**
CRB-65	0.85 ± 0.07	0.7 ± 0.08	0.6 ± 0.09			1.2 ± 0.11	**0.001**
PSI	65.8 ± 2.53	57.8 ± 3.52	51.4 ± 3.67			84.8 ± 4.69	**0.012**
SMART-COP	1.7 ± 0.12	1.1 ± 0.16	1.2 ± 0.16			2.5 ± 0.25	**0.001**
APACHE II	7.7 ± 0.51	6.5 ± 0.41	5.2 ± 0.38			10.4 ± 0.88	**0.001**

Data in bold denote statistically significant results

### IL-2 levels in CAP patients of various severity

In contrast to the serum IL-2 levels observed in healthy individuals, the serum IL-2 levels were significantly elevated in CAP patients (Fig. 1A). The levels of IL-2 were compared among CAP patients with various severities. Based on the CURB-65 score, serum IL-2 levels were substantially lower in group 0–1 and 2 than these in group 3–5 (Fig. 1B). According to SMART-COP score, an obvious rise of IL-2 in group 5–6 and 7–8 compared to CAP patients with the group 0–2 and 3–4 scores, and the serum IL-2 levels were much greater in group 7–8 than these in the other groups (Fig. 1C). In the CRB-65 score, the IL-2 levels of the group ≥ 3 scores were higher than that of the group in 0 and the 1–2 scores (Fig. 1D). According to the PSI score, IL-2 gradually increased with the PSI score, with the most pronounced increase in the group V (Fig. 1E). On the basis of APACEH II score, the levels of IL-2 in the group > 9 scores were markedly elevated than the group 4–6 and 6–9 scores (Fig. 1F).

### Relationships between serum IL-2 and clinical features

**Fig. 2:**
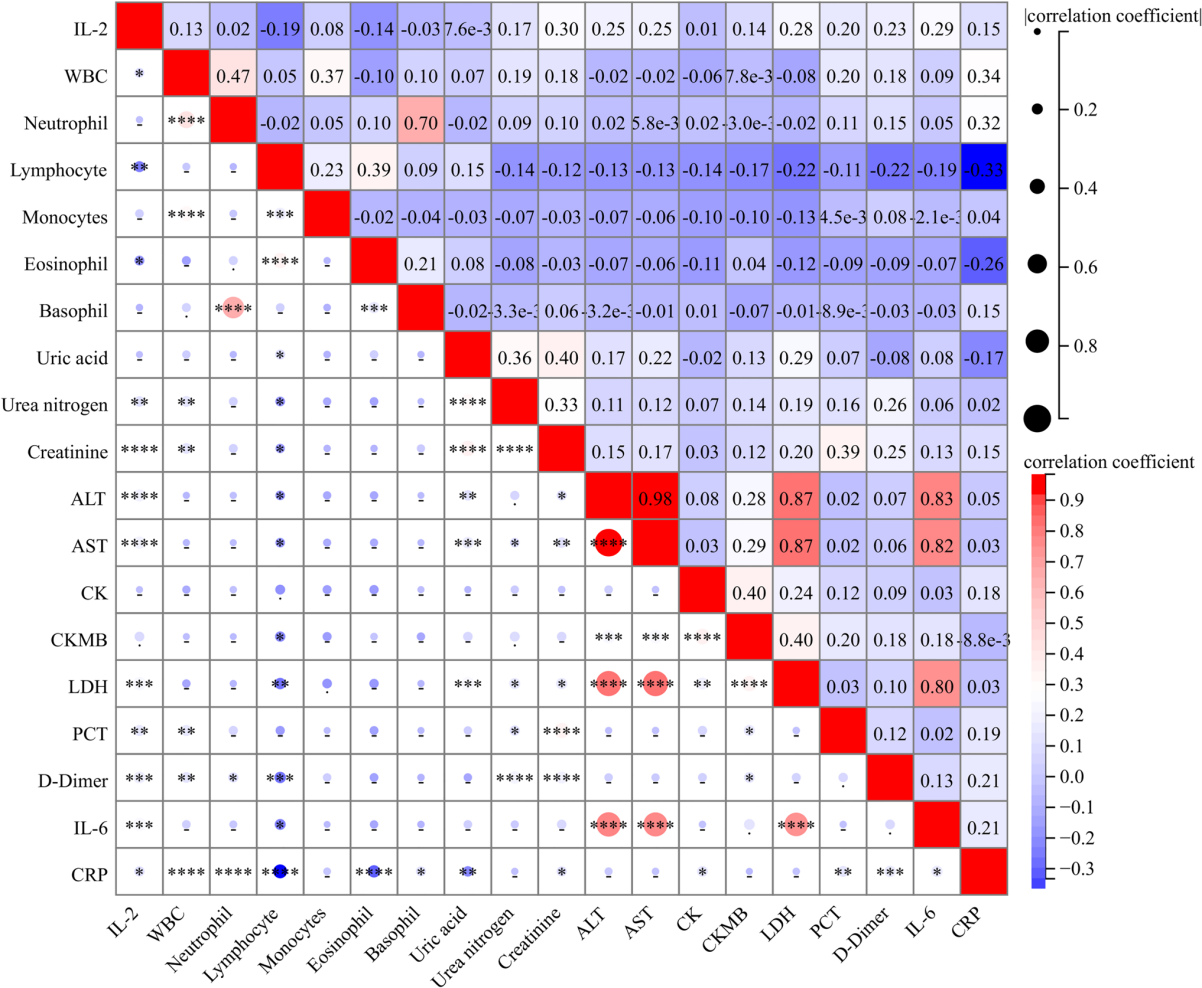
Relationships between serum IL-2 and clinical characteristics in CAP patients. The correlations between serum IL-2 and clinical characteristics were examined by Spearman or Pearson correlative analysis. The top half values indicated the degree of correlation strength. Red color indicates the positive correlation, and blue color indicates the negative correlation. The darker the color, the stronger the correlation. *P < 0.05, **P < 0.01, ***P < 0.001, ****P < 0.0001

In this project, the correlation between serum IL-2 levels and blood routine, liver, and kidney function were evaluated in CAP patients. The data demonstrated a positive correlation between serum IL-2 and white blood cell (WBC) (r = 0.13, *P* < 0.05), a negative relationship between serum IL-2 with lymphocyte (r = -0.19, *P* < 0.01) and eosinophil (r = -0.14, *P* < 0.05). However, there was no meaningful relationship between the level of IL-2 and neutrophil, monocyte, and basophil counts. Additionally, serum IL-2 levels were strongly correlated with aspartate aminotransferase (AST) (r = 0.25, *P* < 0.001), alanine aminotransferase (ALT) (r = 0.25, *P* < 0.001), urea nitrogen (r = 0.17, *P* < 0.01), creatinine (r = 0.3, *P* < 0.001), and lactate dehydrogenase (LDH) (r = 0.28, *P* < 0.001) levels. Moreover, serum IL-2 was positively correlated with D-dimer (r = 0.23, *P* < 0.001), PCT (r = 0.20, *P* < 0.05) (Fig. 2). The mixed linear regression model showed that each 1 pg/mL increase in serum IL-2 was negatively associated with lymphocyte (β = -0.012; 95% CI: -0.021 ~ -0.003) and eosinophil (β = -0.002; 95% CI: -0.004 ~ 0.000). In addition, serum IL-2 were positively correlated with creatinine (β = 1.217; 95% CI: 0.634 ~ 1.800), ALT (β = 5.451; 95% CI: 2.868 ~ 8.034), AST (β = 15.373; 95% CI: 8.090 ~ 22.657), LDH (β = 10.213; 95% CI: 4.789 ~ 15.637), PCT (β = 0.122; 95% CI: 0.026 ~ 0.219), D-dimer (β = 0.049; 95% CI: 0.015 ~ 0.083), IL-6 (β = 6.812; 95% CI: 3.029 ~ 10.595) among CAP patients (Supplemental Table 1).

### Relationships between serum IL-2 and CAP severity scores

The mixed linear and logistic regression models were adjusted for age, hypertension, diabetes mellitus, cerebral infarction, coronary heart disease, and bronchitis. The mixed linear regression model found that each 1 pg/mL increase in serum IL-2 was associated with 0.028 scores (95% CI: 0.016 ~ 0.041), 0.040 scores (95% CI: 0.025 ~ 0.054), 0.094 scores (95% CI: 0.068 ~ 0.120), and 0.357 scores (95% CI: 0.284 ~ 0.431) increases for CRB-65, CURB-65, SMART-COP, and APACHE II, respectively (Table 2). In addition, mixed logistic regression models were performed. Compared to the lowest IL-2 group (T1), the highest IL-2 group (T3) in CAP patients showed a 6.650-fold increase in CRB-65, a 4.860-fold increase in CURB-65, a 2.644-fold increase in SMART-COP, and a 1.989-fold increase in APACHE II. The above results displayed the positive correlations between serum IL-2 levels and the CAP severity scores (Table 2).

**Table 2:** Associations between serum IL-2 and CAP severity scores

Variables	Estimated changes continuous serum IL-2	Estimated changes (95% CI) by tertiles of serum IL-2			*P* trend
		Tertile 1	Tertile 2	Tertile 3	
N	267	89	89	89	
CRB-65	**0.028 (0.016, 0.041)**	0 (Ref)	1.709 (0.659, 4.433)	**6.650 (2.863, 15.445)**	** < 0.001**
CURB-65	**0.040 (0.025, 0.054)**	0 (Ref)	1.041 (0.450, 2.408)	**4.860 (2.295, 10.292)**	** < 0.001**
SMART-COP	**0.094 (0.068, 0.120)**	0 (Ref)	1.401 (0.681, 2.880)	**2.644 (1.335, 5.237)**	**0.004**
PSI	**1.554 (1.051, 2.057)**	0 (Ref)	0.774 (0.369, 1.625)	1.868 (0.897, 3.893)	0.138
APACHE II	**0.357 (0.284, 0.431)**	0 (Ref)	0.679 (0.335, 1.373)	**1.989 (1.003, 3.939)**	0.061

Age, hypertension, diabetes mellitus, cerebral infarction, coronary heart disease, and bronchitis were adjusted

Data in bold denote statistically significant results

### Relationships between serum IL-2 and prognostic outcomes

Throughout the duration of hospitalization, poor prognosis was carefully observed and meticulously tracked in CAP patients (Table 3). Compared with T1 group, mechanical ventilation was observed in 31 patients (34.8%) (RR = 7.753; 95% CI: 2.940 ~ 20.447), 17 patients (19.1%) received vasoactive agent (RR = 8.983; 95% CI: 1.757 ~ 20.372), there were 33 patients (37.1%) with ICU admission (RR = 7.576; 95% CI: 2.858 ~ 20.085), 18 patients dead in-hospital (20.2%) (RR = 13.137; 95% CI: 2.685 ~ 64.267), and 35 patients (39.3%) with longer hospital stays (RR = 3.708; 95% CI: 1.719 ~ 7.995) in T3 group (Table 3). The numbers of the poor prognosis in CAP patients were much higher in T3 group compared to the other groups. These results showed that an elevated serum IL-2 on admission was positively associated with an increased risk of mortality in-hospital, ICU admission, mechanical ventilation, vasoactive drugs and length of hospitalization in CAP patients.

**Table 3:** Relative risk for prognostic outcomes by tertiles of serum IL-2

Variables	Serum IL-2			*P*trend
	Tertile 1	Tertile 2	Tertile 3	
N	89	89	89	
Mechanical ventilation				
N, (%)	7 (7.9)	4 (4.5)	**31 (34.8)**	** < 0.001**
RR (95% CI)	Ref (1)	0.684 (0.182, 2.575)	**7.753 (2.940, 20.447)**	** < 0.001**
Vasoactive agent				
N, (%)	4 (4.5)	3 (3.4)	**17 (19.1)**	**0.001**
RR (95% CI)	Ref (1)	0.862 (0.173, 4.291)	**5.983 (1.757, 20.372)**	**0.005**
ICU admission				
N, (%)	7 (7.9)	4 (4.5)	**33 (37.1)**	** < 0.001**
RR (95% CI)	Ref (1)	0.622 (0.163, 2.372)	**7.576 (2.858, 20.085)**	**0.008**
Death				
N, (%)	1 (1.1)	1 (1.1)	**18 (20.2)**	** < 0.001**
RR (95% CI)	Ref (1)	0.789 (0.356, 2.236)	**13.137 (2.685, 64.267)**	**0.011**
Longer hospital stays				
N, (%)	12 (13.5)	8 (9.0)	**35 (39.3)**	**0.012**
RR (95% CI)	Ref (1)	0.707 (0.267, 1.874)	**3.708 (1.719, 7.995)**	**0.033**

Age, hypertension, diabetes mellitus, cerebral infarction, coronary heart disease, and bronchitis were adjusted

The length of hospital stay was divided into two groups: longer hospital stays, ≥ 14 days; lower hospital stays, < 14 days

*RR* Relative risk

Data in bold denote statistically significant results

### The predictive capacities for severity and death between serum IL-2 and clinical characteristics

**Fig. 3:**
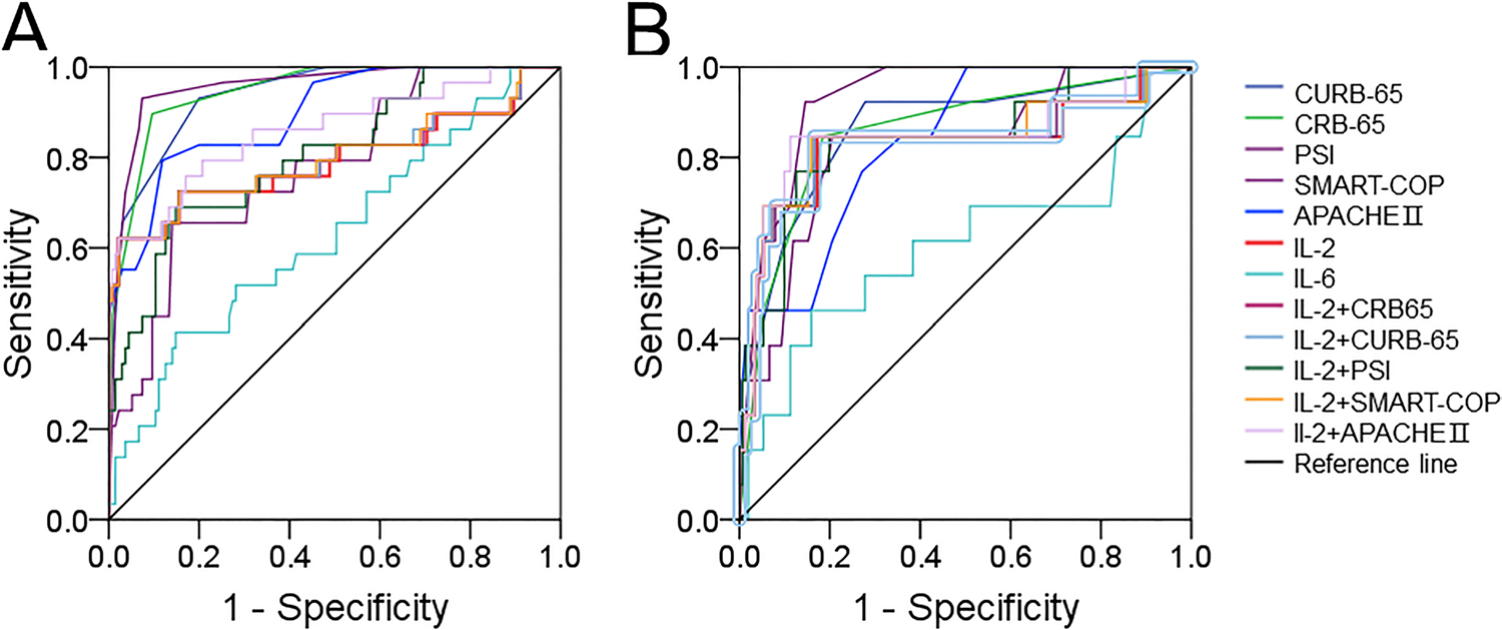
Predictive powers for severity and death in CAP patients. The predictive abilities for severity and death were estimated by ROC curve among CAP patients. **A** ROC curve was used to evaluate the severity. **B** ROC curve was used to evaluate the death risk

The estimation of the predictive ability for severity and mortality were conducted through the receiver operating characteristic (ROC) area under the curve (AUC). The predicted capacities for severities were as follows: CURB-65, 0.898; CRB-65, 0.915; PSI, 0.842; SMART-COP, 0.947; APACHE II, 0.842; IL-2, 0.702; IL-6, 0.663; IL-2 + CRB-65, 0.706; IL-2 + SMART-COP, 0.708; IL-2 + CURB-65, 0.707; IL-2 + PSI, 0.858; IL-2 + APACHE II, 0.755 (Fig. 3A). The cut-off concentration of serum IL-2 was 10.40 pg/mL. The precision of the assessment was determined, exhibiting a sensitivity of 69% and a specificity of 97%. In addition, the capability of predicting mortality was also examined. The levels of predictability were as follows: serum IL-2, 0.817; CRB-65, 0.858; SMART-COP, 0.944; CURB-65, 0.865; APACHE II, 0.849; PSI, 0.831; IL-2 + CRB-65, 0.817; IL-2 + SMART-COP, 0.819; IL-2 + CURB-65, 0.818; IL-2 + PSI, 0.844; IL-2 + APACHE II, 0.840; IL-6, 0.580 (Fig. 3B). The threshold of serum IL-2 for mortality was 8.50 pg/mL, exhibiting a specificity of 82% and a sensitivity of 90%.

## Discussion

This prospective cohort study was aimed to explore the relationships between serum IL-2 levels with the severity and prognosis of CAP patients. The key findings included: (1) there were the positive correlations between the CAP severity scores and the levels of serum IL-2; (2) the risk of poor prognosis was positively associated with the elevated serum IL-2 levels on admission in CAP patients; (3) serum IL-2 had a certain predictive ability for the severity and mortality of CAP.

In the human organism, IL-2 is predominantly released by activated T lymphocytes, while dendritic cells and macrophages also possess the capacity to secrete limited quantities of IL-2 [29]. The effects of IL-2 are contingent upon its dosage and affinity for receptors, leading to a diverse range of outcomes [30–33]. Published research studies have shown that IL-2 is strongly connected to the biological processes of many lung diseases. In an experimental model utilizing mice, IL-2 is significantly elevated and can exacerbate allergic asthma through the toll-like receptor 9-IL-2 axis [34]. Patients with COVID-19 who are undergoing cytokine storm demonstrate elevated serum levels of IL-2, which serves as an immunopathologic attribute [35–37]. In addition, IL-2 can synergistically promote vascular leakage with TNF-α in superantigen or pathogen-induced acute lung injury [20, 38]. However, the exact connection between IL-2 and CAP has not yet been identified. Therefore, IL-2 levels were examined in all CAP patients. Based on our study, we discovered that patients with CAP had gradually rising serum IL-2 levels as their severity scores rose. Furthermore, serum IL-2 levels on admission were strongly connected to CAP severity scores. Additionally, various forms of organ impairment were found in CAP patients [22–26]. Therefore, we estimated the relationships between serum IL-2 and various clinical parameters. The facts from our study revealed that serum IL-2 were positively correlated with WBCs, other inflammatory factors, and indices related to hepatic and renal functions, such as urea nitrogen, creatinine, AST, ALT. The implications of these findings propose that IL-2 may play a pivotal role in the pathophysiological mechanisms of CAP.

Numerous investigations have substantiated the findings that IL-2 is involved in the progression of many other diseases and associated with its prognosis. High expression of IL-2 is positively correlated with the survival period of multiple myeloma patients [39]. The presence of IL-2 exacerbates the poor prognosis of colorectal cancer patients [40]. When IL-2 is elevated in patients diagnosed with non-Hodgkin lymphoma, their survival rates are diminished [41]. But the relationship between IL-2 and the prognosis was unclear in CAP patients. Our research revealed that the increased IL-2 levels on admission elevated the risks of the mortality in-hospital, ICU admission, mechanical ventilation, vasoactive drugs, and longer hospital stays in CAP patients. The predictive abilities of serum IL-2, other inflammatory markers, and severity scores for severity and mortality were assessed through ROC curve. The results indicated that IL-2 had a certain predictive ability for the severity and mortality of CAP. Although the predictive capacities of serum IL-2 for severity and death were slightly weaker than those in CAP severity scores system. They were obviously elevated compared with commonly inflammatory cytokines, such as IL-6. Even so, serum IL-2 combination with CAP severity scores can’t elevated the predive powers for severity and death compared with single serum IL-2 or CAP severity scores among CAP patients. These results suggested that IL-2 is implicated in the pathophysiological processes of CAP. It is possible that IL-2 inhibition can decelerate the progression of illness in CAP. Therefore, serum IL-2 level may be used as a candidate biomarker for guiding clinical practice, including illness estimate, prognose analysis, and therapeutic targets. Thus, the results from our study demonstrated that higher levels of serum IL-2 on admission were associated with an increased risk of poor prognosis in CAP patients.

IL-2 is recognized to be a common pro-inflammatory factor that is widely expressed in human immune cells [29]. CAP can be caused by various pathogenic infections, mainly included gram-positive bacteria, gram-negative bacteria, viruses and other atypical pathogens [42]. Gram-negative bacterial cell membranes contain lipopolysaccharide. When it binds to TLR4 expressed on airway epithelial cells [43], it activates the NF-κB transcription factor, causing pro-inflammatory cytokines like IL-2, IL-4, IL-5, and IL-6 to be released [44]. Additionally, SARS-CoV-2 infection can activate NF-κB, resulting in a "cytokine storm" that releases plenty of inflammatory factors, including IL-2, which in turn leads to lung damage in COVID-19 patients [45]. High IL-2 concentration can induce vascular leakage syndrome and cytokine storms, leading to interstitial pulmonary edema and multi-organ failure [46–48]. An animal experiment indicated that Streptococcus pneumonia infection incurs pulmonary inflammation and IL-2 elevation in the lungs [49]. Therefore, we speculate that pathogenic infection may activate NF-κB signaling and evoke inflammatory cytokines secretion, leading to IL-2 elevation in lungs. Then, IL-2 is secreted, and the levels of serum IL-2 are elevated in CAP patients.

This research endeavored to illuminate the role of IL-2 in CAP. It predominantly demonstrates the positive correlations between serum IL-2 concentration on admission and the severity as well as unfavorable prognosis of CAP patients. Nevertheless, the present investigation possessed certain limitations. First, as a result of the limited sample size at present, future research necessitates larger sample sizes and the inclusion of multiple centers. Second, only serum samples were utilized to assess IL-2 levels. Further investigation is warranted to assess the levels of IL-2 in pulmonary tissues and bronchoalveolar lavage fluid. Third, the current study only was an epidemiological examination of the population. The mechanism underlying the enhancement in IL-2 remained unidentified in CAP patients. Animal experiments are required to perform and explore the exact mechanism.

In the previous studies, CAP patients are enrolled from inpatient department. Actually, most CAP patients are treated and cured in the outpatient department [50]. Due to the disease condition, there is no need to be hospitalized for the majority of CAP cases. So, the conclusions are extrapolated form inpatients to outpatients. This is the current research status. Mounting evidence have revealed that mild CAP patients can be treated and cured in the outpatient department. The mortality rate is very low (0.5%), and it is evidently lower than those in the current research (7.49%). Moreover, the risk of poorly prognostic outcomes is infrequent among CAP patients [51]. Therefore, we think it doesn’t affect the validity of the extrapolation of the results. Of course, this is a research defect. To improve the reliability of conclusions, more confirmed research will be conducted in the outpatients.

## Conclusions

Through a prospective cohort analysis, this research verified the relationships between serum IL-2 and the severity and prognostic outcomes of CAP patients. We discovered that serum IL-2 levels were gradually increased with elevated CAP severity scores. There were strong associations between serum IL-2 levels and clinical parameter in CAP patients. In addition, Serum IL-2 were positively related to CAP severity scores and poor outcomes. All conclusions suggest that IL-2 may involve in the pathophysiological process of CAP. Therefore, in forthcoming clinical practice, the potential of serum IL-2 as a valuable biomarker for assessing the severity and prognosis is anticipated to be explored in CAP patients.

## Supplementary Information

Below is the link to the electronic supplementary material.

Supplementary file1 (DOCX 166 KB)

Supplementary file2 (DOC 48 KB)

## Data Availability

The corresponding author can provide all the data and information of this research.
