# Supplementary figures and images for: Association of serum interleukin-2 with severity and prognosis in hospitalized patients with community-acquired pneumonia: a prospective cohort study
Source: Intern Emerg Med. 2024 Jul 5;19(7):1929–39. doi: 10.1007/s11739-024-03699-0 (PMC11467086; doi:10.1007/s11739-024-03699-0)

**Supplemental Fig. 1 Flow diagram of recruitment and follow-up research in this cohort study.**


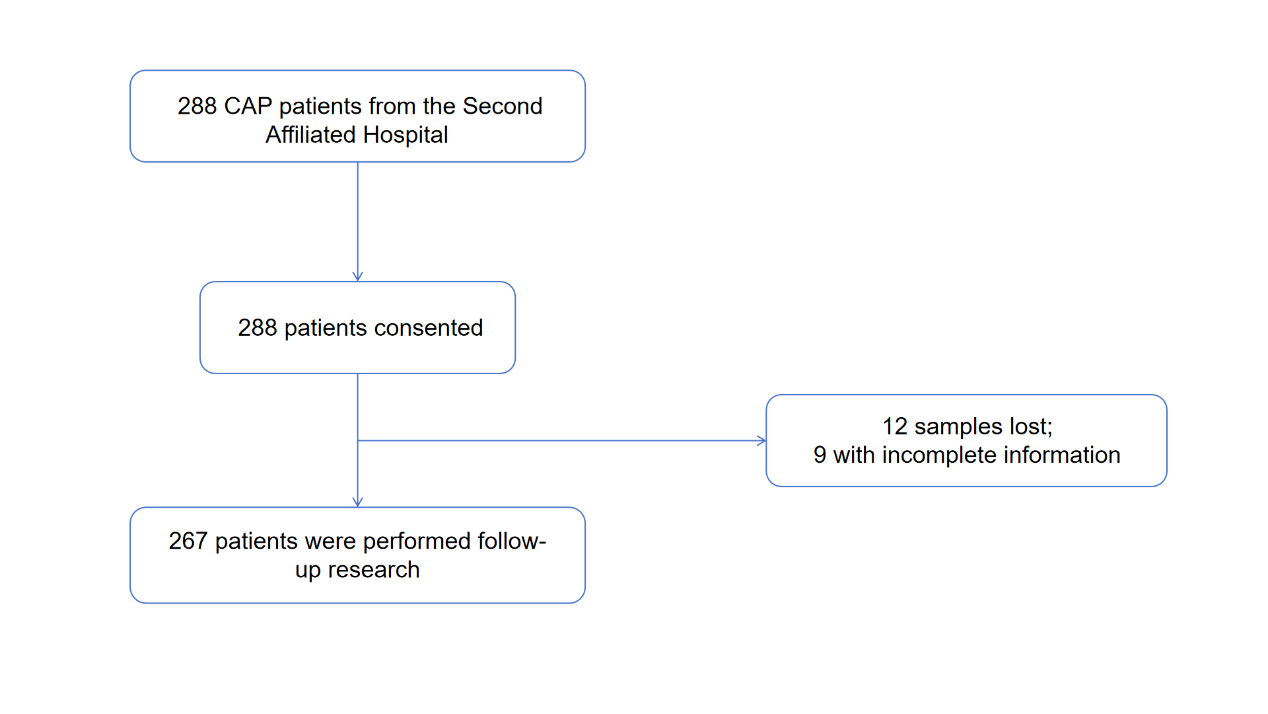

Supplement: Supplementary file 1 — Supplementary file1 (DOCX 166 KB) [file 11739_2024_3699_MOESM1_ESM.docx]
