# Supplementary material for: Association of serum interleukin-2 with severity and prognosis in hospitalized patients with community-acquired pneumonia: a prospective cohort study
Source: Intern Emerg Med. 2024 Jul 5;19(7):1929–39. doi: 10.1007/s11739-024-03699-0 (PMC11467086; doi:10.1007/s11739-024-03699-0)
Supplement: Supplementary file 2 — Supplementary file2 (DOC 48 KB) [file 11739_2024_3699_MOESM2_ESM.doc]

Supplemental Table 1. Associations between serum IL-2 and CAP severity scores.

| Variables | Estimated changes continuous serum IL-2 | *P* |
| --- | --- | --- |
| N | 267 |  |
| WBC | 0.084 (-0.019, 0.102) | 0.181 |
| Neutrophil | 0.012 (-0.152, 0.184) | 0.854 |
| Lymphocyte | -0.012 (-0.021, -0.003) | **0.010** |
| Monocyte | 0.082 (-0.002, 0.011) | 0.204 |
| Eosinophil | -0.002 (-0.004, 0.000) | **0.016** |
| Basophil | -0.006 (-0.017, 0.016) | 0.930 |
| Uric acid | 0.008 (-1.705, 1.947) | 0.897 |
| Urea nitrogen | 0.093 (-0.025, 0.193) | 0.129 |
| Creatinine | 1.217 (0.634, 1.800) | **＜0.001** |
| ALT | 5.451 (2.868, 8.034) | **＜0.001** |
| AST | 15.373 (8.090, 22.657) | **＜0.001** |
| CK | 2.568 (-9.031, 14.167) | 0.662 |
| CKMB | 0.184 (-0.036, 0.405) | 0.100 |
| LDH | 10.213 (4.789, 15.637) | **＜0.001** |
| PCT | 0.122 (0.026, 0.219) | **0.013** |
| D-Dimer | 0.049 (0.015, 0.083) | **0.005** |
| IL-6 | 6.812 (3.029, 10.595) | **＜0.001** |
| CRP | -0.542 (-2.053, 0.969) | 0.480 |

Age, hypertension, diabetes mellitus, Cerebral infarction, and coronary heart disease were adjusted.

Data in bold denote statistically significant results.
